# Treat-and-extend therapy using intravitreal aflibercept for neovascular age-related macular degeneration: 2-year real-world practice data from Slovenia

**DOI:** 10.1186/s12886-018-1005-x

**Published:** 2018-12-20

**Authors:** Polona Jaki Mekjavić, Bogdan Gregorčič, Cvetka Oberč, Slava Podgoršek

**Affiliations:** 1 Eye Hospital, University Medical Center Ljubljana, Grabloviceva 46, SI-1000 Ljubljana, Slovenia; 2 Medical Faculty, University of Ljubljana, Ljubljana, Slovenia; 3 Eye Department, General Hospital Dr. Franca Derganca, Nova Gorica, Slovenia; 4 Eye Department, General Hospital, Novo Mesto, Slovenia; 5 Eye Department, General Hospital, Celje, Slovenia

**Keywords:** Aflibercept, Anti-VEGF, Neovascular age-related macular degeneration, Real-world data, Treat and extend, Visual acuity

## Abstract

**Background:**

To assess visual outcomes over 24 months in patients with neovascular age-related macular degeneration (nAMD) who initiated intravitreal aflibercept therapy under a treat-and-extend (TE) regimen in real-world settings.

**Methods:**

In this retrospective, observational, multicentre study in Slovenia, medical records of all treatment-naïve patients with nAMD who started intravitreal aflibercept therapy between October 2013 and April 2015 were reviewed. The primary outcome measure was change in mean visual acuity (VA) from baseline to 24 months in patients who received the TE regimen for 2 years, assessed by standardised Early Treatment Diabetic Retinopathy Study charts and calculated as least-squares means. Other outcome measures included the numbers of injections and visits at 12 months and 24 months.

**Results:**

The primary analysis included 115 eyes of 105 patients who received TE treatment for 2 years (Group A). The mean VA improved from 57.9 ± 14.9 letters at baseline to 64.6 ± 15.8 letters (+ 6.5 letters, *p* < 0.0001) at 12 months and 64.8 ± 15.6 letters (+ 7.0 letters, p < 0.0001) at 24 months. The mean number of injections per eye was 8.4 ± 1.9 and the mean number of visits was 8.8 ± 1.7 at 12 months; these numbers decreased to 6.1 ± 2.0 and 6.4 ± 1.9, respectively, at 24 months. The additional analysis included 33 eyes of 33 patients who received TE treatment in Year 1, followed by pro re nata treatment in Year 2 (Group B). Compared with Group A whose vision improvement was maintained at 24 months, the VA gain in Group B eyes seen at 12 months (change in mean VA vs baseline: + 6.9 letters, *p* = 0.0008) was no longer present at 24 months (change in mean VA vs baseline: + 1.2 letters, *p* = 0.5733).

**Conclusions:**

Using the TE regimen in clinical practice, intravitreal aflibercept significantly improved visual outcomes in treatment-naïve patients with nAMD, which were maintained over time. TE therapy with intravitreal aflibercept is a rational long-term strategy that can produce favourable outcomes in clinical practice.

## Background

Age-related macular degeneration (AMD) is one of the leading causes of visual impairment in industrialised countries [1]. The advanced stage of the disease, neovascular AMD (nAMD), is responsible for the most severe vision loss and can have a debilitating effect on quality of life [2, 3]. The unravelling of the core molecular mechanisms of nAMD has led to the approval of two robust anti-vascular endothelial growth factor (VEGF) therapies which have now become the standard of care [4, 5]. Findings from the pivotal ranibizumab studies ANCHOR and MARINA [6, 7], and aflibercept studies VIEW 1 and VIEW 2 [8, 9], have shown clinical benefits of these anti-VEGF agents in improving visual acuity (VA), preventing loss of vision or maintaining vision in most patients with nAMD.

In addition to the fixed-dosing protocol during the first year as per label [4], pro re nata (PRN) and treat-and-extend (TE) are also used for intravitreal aflibercept injections in clinical practice [10]. Initially described by Spaide in 2007 [11], the individually tailored TE approach is gaining popularity among retinal specialists to minimise the need for frequent treatment and the burden on patients [12]. Due to its earlier availability, ranibizumab treatment using a TE strategy for nAMD has been investigated in both randomised controlled trials (RCTs) and real-world studies, and has been reported to be comparable with fixed-dosing regimens and either equivalent or superior to PRN protocols [13–17]. However, limited information is available on TE approaches in nAMD using intravitreal aflibercept: a small prospective trial and a retrospective database study provide evidence that intravitreal aflibercept TE therapy can produce good outcomes over 2 years while reducing the treatment burden [18, 19].

Intravitreal aflibercept was first registered in Slovenia in November 2012 [4] and was used for the treatment of nAMD in four centres in the country at the time of this study. This retrospective analysis reported the 24-month VA outcomes in treatment-naïve patients with nAMD using intravitreal aflibercept in routine clinical practice from all four centres in Slovenia. The primary objective of the study was to assess visual outcomes over 24 months in patients who received intravitreal aflibercept therapy under a TE regimen.

## Methods

A retrospective, observational, multicentre study of nAMD patients in Slovenia over a 2-year period was conducted after requesting the approval of the National Medical Ethics Committee of Slovenia. We retrospectively identified in our medical records all patients who started treatment with intravitreal aflibercept between October 2013 and April 2015 for the indication of treatment-naïve nAMD in at least one eye. Patient documentation was reviewed and data collected at 4, 12, 18 and 24 months. In total, four sites in Slovenia participated in this study: Eye Hospital University Medical Center Ljubljana, General Hospital “Dr Franca Derganca” Nova Gorica, General Hospital Nova Mesto, and General Hospital Celje. All patients included in this study provided written informed consent to the use of their anonymised data for the purposes of clinical audit and research, as per participating hospital policy.

We reviewed a series of consecutive cases of 166 patients (182 eyes) with active nAMD who were offered treatment with intravitreal aflibercept. Diagnosis was confirmed by clinical examination (slit-lamp fundoscopy), spectral domain optical coherence tomography (SD-OCT) and angiography. Inclusion criteria included treatment-naïve active nAMD, proven/diagnosed with fluorescein and/or indocyanine green angiography. Different types of active lesions of all sizes were included: classic, occult, minimally classic and retinal angiomatous proliferation; polypoid lesions were excluded.

Patients received intravitreal aflibercept following one of the three protocols: 1) TE for 2 years; 2) TE for 1 year and then switching to PRN the following year; 3) PRN for 2 years. All patients received consecutive injections of intravitreal aflibercept at 4- to 5-week intervals until an anatomical improvement of the macula was apparent, as noted by SD-OCT as a reduction in intraretinal and subretinal fluid. When the macula became dry, in patients following the TE protocol, there was a stepwise prolongation of treatment intervals of 2 weeks up to 14 weeks. If a recurrence of subretinal and/or intraretinal fluid or new haemorrhage was evident, the treatment interval was reduced by 2 weeks. Patients were examined and treated on the same day. Patients following the PRN protocol had bimonthly follow-up and received additional reinjections if any of the following changes were observed by the evaluating physician as shown: (1) VA loss of at least 5 letters with SD-OCT evidence of subretinal and/or intraretinal fluid, (2) new macular haemorrhage, or (3) evidence of persistent/increased subretinal and/or intraretinal fluid on SD-OCT at least 1 month after the previous injection. All retreatment criteria were based by comparing the results with the previous visit.

A shared decision-making approach based on both the patients’ and the treating ophthalmologists’ preferences was used to determine the treatment protocol before treatment initiation and during the annual review. The TE protocol was used if receiving treatment on each visit with possible extension of treatment intervals was preferred, whereas PRN was used if more frequent monitoring and less injections were preferred.

On each visit, patients were questioned about any adverse events (AEs) observed between visits. VA was measured by standardised Early Treatment Diabetic Retinopathy Study (ETDRS) charts; SD-OCT (Topcon or Heidelberg) and slit-lamp fundoscopy were performed.

The primary analysis included eyes that received TE treatment for 2 years (Group A) and the primary measure was change in mean VA from baseline to 24 months. Other measurements included the mean number of injections per eye, the mean number of visits and the proportions of eyes stratified by VA at 4, 12 and 24 months. Additional analysis included comparison of VA outcomes between Group A eyes and those switched from the TE to PRN regimen in Year 2 (Group B).

### Statistical analysis

Calculations were performed with SAS Version 9.4 (SAS Institute Inc., Cary, NC, USA).

If not otherwise stated, all values are presented as mean ± standard deviation. VA measurements were analysed using mixed models with repeated measures to account for the availability of two measurements (both eyes) for some patients. The VA at a given time point was modelled as a dependent variable, while baseline VA was used as an independent variable. An unstructured covariance structure was chosen and the patients constituted a repeated factor. To investigate the two different treatment regimens, the regimen was used as an additional fixed factor in an extended model. Least-squares means and their 95% confidence intervals were utilised to estimate mean absolute values, mean absolute changes and mean differences in changes. *P*-values of the corresponding type 3 tests of fixed effects were considered as statistically significant when they were below 0.05.

## Results

### Baseline characteristics

The final analysis included 115 eyes of 105 patients who received TE treatment for 2 years (Group A) and 33 eyes of 33 patients who received TE treatment for 1 year and PRN treatment for the following year (Group B), excluding 30 eyes of 24 patients treated with PRN aflibercept for 2 years and 4 eyes of 4 patients that were lost to follow-up. The baseline characteristics of all patients included in the study are summarised in Table 1. The demographic characteristics between Group A and Group B eyes were comparable.

**Table 1:** Patient demographics and baseline characteristics

	Group A	Group B	p-value
Number of patients	105	33	
Number of eyes	115	33	
Female/Male	67/38	20/13	0.8367^a^
Age (years, mean ± SD)	77.3 ± 7.3	76.3 ± 8.4	0.5303^b^
VA (by eye, mean ± SD)	57.9 ± 14.9	62.4 ± 12.4	0.0850^b^

^a^Fisher’s exact test; ^b^ t-test assuming unequal variances (Satterthwaite method)

Group A: eyes receiving the TE regimen for 2 years; Group B: eyes receiving the TE regimen in Year 1 and the PRN regimen in Year 2. PRN: pro re nata; SD: standard deviation; TE: treat-and-extend; VA: visual acuity

### Primary analysis

**Fig. 1:**
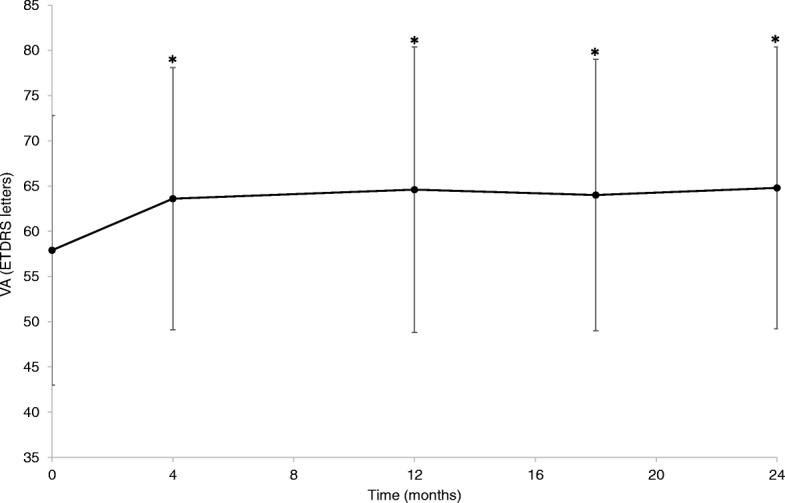
Mean VA ± SD from baseline to 24 months in eyes receiving treat-and-extend aflibercept for 2 years (*n* = 115) *p < 0.05. VA values refer to least-squares means. ETDRS: Early Treatment Diabetic Retinopathy Study; SD: standard deviation; VA: visual acuity

**Fig. 2:**
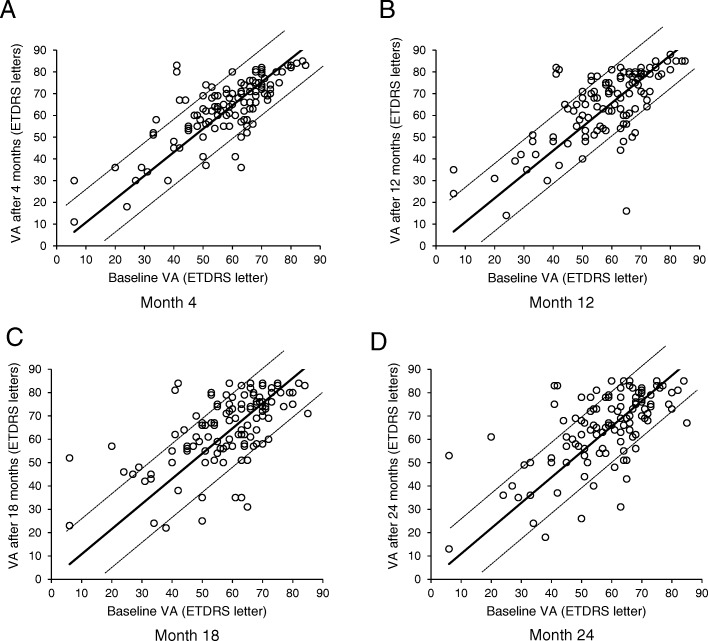
Scatterplots of baseline VA vs VA at 4, 12, 18 and 24 months in eyes receiving treat-and-extend aflibercept for 2 years (*n* = 115) Points above the upper / below the lower dotted lines represent a gain / loss, respectively, of more than 15 ETDRS letters. ETDRS: Early Treatment Diabetic Retinopathy Study; VA: visual acuity

The mean VA over the course of the study in Group A eyes is illustrated in Fig. 1. Scatterplots of VA at baseline vs VA at 4, 12, 18 and 24 months are shown in Fig. 2 A–D, respectively. The mean VA and change in mean VA vs baseline are detailed in Table 2. After 12 months, 48.7% of eyes had VA of ≥70 letters (Table 3); the mean number of injections per eye was 8.4 ± 1.9 and the mean number of visits was 8.8 ± 1.7. After 24 months, 43.5% of eyes had VA of ≥70 letters; the mean number of injections per eye and the mean number of visits decreased to 6.1 ± 2.0 and 6.4 ± 1.9, respectively.

**Table 2:** Mean VA and change in mean VA from baseline at 4, 12, 18 and 24 months

	Group A	Group B
Number of patients	105	33
Number of eyes	115	33
	Baseline	
VA (mean ± SD)	57.9 ± 14.9	62.4 ± 12.4
*p*-value	0.0850	
	4 months	
VA (mean ± SD)	63.6 ± 14.5	66.8 ± 12.4
Change in mean VA (95% CI)	5.5	5.4
95% CI	[3.7; 7.2]	[2.0; 8.7]
*p*-value	< 0.0001	0.0017
Difference in mean VA changes	0.1	
95% CI	[−3.7; 3.9]	
*p*-value	0.9619	
	12 months	
VA (mean ± SD)	64.6 ± 15.8	68.5 ± 14.4
Change in mean VA (95% CI)	6.5	6.9
95% CI	[4.4; 8.6]	[3.0; 10.9]
*p*-value	< 0.0001	0.0008
Difference in mean VA changes	−0.5	
95% CI	[−5.0; 4.0]	
*p*-value	0.8378	
	18 months	
VA (mean ± SD)	64.0 ± 15.0	65.8 ± 15.5
Change in mean VA (95% CI)	6.0	4.3
95% CI	[3.7; 8.3]	[0.1; 8.6]
*p*-value	< 0.0001	0.0467
Difference in mean VA changes	1.7	
95% CI	[−3.1; 6.5]	
*p*-value	0.4952	
	24 months	
VA (mean ± SD)	64.8 ± 15.6	63.2 ± 17.3
Change in mean VA (95% CI)	7.0	1.2
95% CI	[4.7; 9.3]	[−3.1; 5.6]
*p*-value	< 0.0001	0.5733
Difference in mean VA changes	5.7	
95% CI	[0.8; 10.6]	
*p*-value	0.0224	

Group A: eyes receiving the TE regimen for 2 years; Group B: eyes receiving the TE regimen in Year 1 and the PRN regimen in Year 2. VA values refer to least-squares means. CI: confidence interval; PRN: pro re nata; SD: standard deviation; TE: treat-and-extend; VA: visual acuity

**Table 3:** Subgroups of eyes according to VA at baseline, 4, 12 and 24 months

VA, letters	Group A (*n* = 115)				Group B (*n* = 33)			
	Baseline	4 months	12 months	24 months	Baseline	4 months	12 months	24 months
	n (%)	n (%)	n (%)	n (%)	n (%)	n (%)	n (%)	n (%)
≥70	24 (20.9)	45 (39.1)	56 (48.7)	50 (43.5)	9 (27.3)	19 (57.6)	19 (57.6)	17 (51.5)
55–69	51 (44.3)	47 (40.9)	29 (25.2)	40 (34.8)	17 (51.5)	7 (21.2)	8 (24.2)	5 (15.2)
35–54	30 (26.1)	17 (14.8)	25 (21.7)	21 (18.3)	6 (18.2)	6 (18.2)	5 (15.2)	9 (27.3)
< 35	10 (8.7)	6 (5.2)	5 (4.3)	4 (3.5)	1 (3.0)	1 (3.0)	1 (3.0)	2 (6.1)

n: number of eyes. Group A: eyes receiving the TE regimen for 2 years; Group B: eyes receiving the TE regimen in Year 1 and the PRN regimen in Year 2. PRN: pro re nata; TE: treat-and-extend; VA: visual acuity

### Additional analysis

The mean VA and change in mean VA vs baseline in Group B eyes at 4, 12, 18 and 24 months are shown in Table 2. After 12 months, 57.6% of eyes had VA of ≥70 letters; the mean number of injections per eye was 7.8 ± 1.4 and the mean number of visits was 8.1 ± 1.4. After 24 months, 51.5% of eyes had VA of ≥70 letters; the mean number of injections per eye and the mean number of visits decreased to 2.5 ± 1.7 and 6.2 ± 1.8, respectively.

When comparing Group B with Group A, the differences in mean VA change from baseline to 4 months and to 12 months were negligible. At 18 months, there was a small, non-statistically significant difference in mean VA change from baseline (1.7 letters, *p* = 0.4952) favouring Group A; this difference increased to 5.7 letters with statistical significance at 24 months (*p* = 0.0224).

### Ocular safety

The following ocular-specific AEs were observed: endophthalmitis (*n* = 1, severe; treated with vitrectomy and intravitreal antibiotics); non-infectious inflammation (*n* = 1, severe; treated with local corticosteroids for several days); dehiscence of conjunctiva on the injection site (*n* = 2, both mild; treated with local antibiotic ointment for a few days). Vitreous opacities (bubbles) were anecdotally reported and considered mild. Precise numbers were not tabulated.

## Discussion

Over the past years, researchers and clinicians have been seeking ways to minimise the frequency of treatment for nAMD patients receiving anti-VEGF therapy without compromising visual outcomes [12]. Positive clinical experiences with the proactive TE dosing regimens that allow extension of treatment intervals have fuelled the spread of the protocol among retinal specialists around the world [12, 20]. The Preferences and Trends Survey indicates that almost 80% of US clinicians adhere to the TE approach, as do about 60% of their foreign counterparts [20].

In this retrospective observational study, intravitreal aflibercept therapy initiated under a TE regimen in treatment-naïve patients with nAMD was assessed over a period of 24 months. The mean baseline VA of 57.9 ± 14.9 letters among patients who received TE therapy for 2 years in our study is slightly higher than that reported in other real-world studies [21–23]. This could introduce potential difficulty in demonstrating improvement in vision because, compared with eyes with lower VA at baseline, eyes with higher VA have reduced chance of gaining more but greater potential to lose vision [18]. We observed significant improvements in mean VA after the initial loading dose, which were maintained for the full 24 months of the study. There was a gain of 6.5 letters in mean VA after 1 year of treatment (*p* < 0.0001) and 7.0 letters after 2 years (p < 0.0001). The proportion of eyes with VA of ≥70 letters more than doubled after 12 months, and remained elevated after 24 months. In the natural course of nAMD, patients typically lose ≥3 VA lines after 12 months [24]. Most eyes in our study had stable VA (± 3 VA lines; dotted lines in Fig. 2) after treatment with intravitreal aflibercept under a TE regimen throughout the study period.

There are currently no data from RCTs on the outcomes of intravitreal aflibercept TE treatment for nAMD. In the VIEW studies, a fixed-dosing protocol was mandated during the first year and after this a “capped PRN” approach was used [25]. The 96-week results of the VIEW studies and our 2-year retrospective real-life study show comparable visual function outcomes, despite following different treatment approaches [25].

Intravitreal aflibercept TE therapy was investigated in the prospective Aflibercept Treat and extend for Less frequent Administration Study (ATLAS) which showed a median VA improvement of 7.5 ETDRS letters from baseline to 2 years in 31 treatment-naïve patients with nAMD, with mean numbers of injections of 8.0 and 6.5 during the first and second year, respectively [19]. That is similar to what we observed. Real-world data of intravitreal aflibercept TE therapy in a larger patient cohort have recently become available – the Fight Retinal Blindness Study Group has found a gain of 6.0 letters in mean VA from 136 eyes of 123 nAMD patients completing 24 months of follow-up in routine clinical practice [18]. The gain of 7.0 letters in mean VA in patients who received TE intravitreal aflibercept for 2 years in our study was slightly higher than the 6.0-letter gain observed by the Fight Retinal Blindness Study Group [18]. This may be in part attributed to the small difference in treatment frequency: the mean numbers of injections in the first and second year were 8.4 and 6.1 in our study, compared with 7.8 and 5.7 reported by the Fight Retinal Blindness Study Group [18].

Our study also provides real-world evidence demonstrating the long-term advantages of the TE approach over PRN in aflibercept-treated nAMD patients. There was no difference in mean VA gain at the end of the loading phase and at 12 months between treatment groups when the TE approach was used in all patients. This improvement in mean VA was maintained at 24 months in patients who continued the TE treatment throughout the 2 years, but lost in those who switched to PRN in the second year. Unsurprisingly, the difference in the mean number of injections per eye in Year 2 between the two treatment groups was apparent: 6.1 vs 2.5. It is interesting to note that the changes in the percentage of eyes with VA of ≥70 letters from Year 1 to Year 2 were similar between the two groups. Among these eyes, one eye from Group A lost 18 letters as a result of a retinal pigment epithelial tear and one eye from Group B lost 40 letters due to the development of subfoveal atrophy; the rest of the eyes in both groups had VA changes of < 3 VA lines. The changes in the proportion of eyes with VA of ≤54 letters, however, followed different patterns in different treatment groups. Among those who followed the PRN protocol in the second year, the proportion of eyes with VA of ≤54 letters increased from under one fifth at 12 months to one third at 24 months, suggesting that the decrease in mean VA from Year 1 to Year 2 in these eyes was mainly a result of an increase in the number of eyes with moderate to severe visual impairment. In comparison, in eyes that received the TE treatment throughout the 2 years, the proportion of those with VA of ≤54 letters decreased slowly but steadily from baseline to 24 months, confirming the benefits of intravitreal aflibercept TE therapy in maintaining vision over time.

Our study is important because it provides real-world data from four centres in Slovenia where all intravitreal aflibercept treatments were conducted at the time, which fully represents a countrywide example of nAMD patient management. Based on our promising findings, intravitreal aflibercept is now being adopted in all seven anti-VEGF treatment centres in Slovenia. Additionally, the study results confirm the long-term benefits of intravitreal aflibercept using a TE regimen and add to the growing literature on the TE approach with anti-VEGF therapy which has been predominantly focused on the use of ranibizumab. The retrospective design of our study may also help to alleviate potential investigator bias which can sometimes be present in open-label studies. As with similar studies, the main limitations of our study are the observational and uncontrolled nature of the study design, as well as the inherent difference between baseline characteristics and disease progression in real-life patients compared with those in RCTs.

## Conclusion

In this retrospective clinical study in real-life settings, intravitreal aflibercept treatment initiated under a TE regimen in treatment-naïve patients with nAMD led to significant visual improvement at 12 months, which was maintained at 24 months. TE therapy with intravitreal aflibercept proved to be a rational long-term strategy that can produce favourable outcomes in clinical practice.

## Abbreviations

AE: Adverse event
ETDRS: Early Treatment Diabetic Retinopathy Study
nAMD: Neovascular age-related macular degeneration
PRN: Pro re nata
RCT: Randomised controlled trial
SD: Standard deviation
SD-OCT: Spectral domain optical coherence topography
TE: Treat-and-extend
VA: Visual acuity
VEGF: Vascular endothelial growth factor.
